# Multiferroic La_0.2_Pb_0.7_Fe_12_O_19_ ceramics: Ferroelectricity, ferromagnetism and colossal magneto-capacitance effect

**DOI:** 10.1016/j.dib.2016.11.067

**Published:** 2016-11-24

**Authors:** Guo-Long Tan, Hao-Hao Sheng

**Affiliations:** State Key Laboratory of Advanced Technology for Materials Synthesis and Processing, Wuhan University of Technology, Wuhan 430070, China

**Keywords:** Multiferroics, La_0.2_Pb_0.7_Fe_12_O_19_, Ferroelectricity, Ferromagnetism, Magnetocapacitance

## Abstract

The mutual control of the electric and magnetic properties of a multiferroic solid is of fundamental and great technological importance. In this article, the synthesis procedure of La_0.2_Pb_0.7_Fe_12_O_19_ ceramics was briefly described and the data acquired for the materials characterization is presented. This data article is related to the research article-Acta Mater. 2016, 121, 144 (j.actamat.2016.08.083). Electric polarization hysteresis loop and I-V curve, which help to confirm the ferroelectricity of La_0.2_Pb_0.7_Fe_12_O_19_ ceramics, were presented. Strong magnetic polarization data was also presented. The great variation of the dielectric constants along with the magnetic field has been presented which helped to demonstrat the giant magnetocapacitance of La_0.2_Pb_0.7_Fe_12_O_19_. All the datasets were collected at room temperature. Large ferroelectricity, strong magnetism and colossal magneto-capacitance effect have been all realized in one single phase La_0.2_Pb_0.7_Fe_12_O_19_ at room temperature.

**Specifications Table**

**Table:** 

Subject area	*Physics*
More specific subject area	*Materials physics, functional materials.*
Type of data	*figures*
How data was acquired	*Instruments used- Bruker D8 Advance for XRD measurement, ZT-IA ferroelectric measurement system, Quantum Design physical property measurement system (PPMS), Wayne Kerr 6500B LCR station.*
Data format	*Raw, analyzed, etc*
Experimental factors	*The ceramics were heat treated in oxygen atmosphere at 700 °C for three times and then both surface sides were coated with silver electrode for ferroelectric measurement and magnetic field-dependent capacitance measurement. The powders for magnetic characterization measurement have also been annealed in O*_*2*_*for three times.*
Experimental features	*The magnetocapacitance data was measured by placing the specimen in a space between two magnets. The magnetic field was adjusted by voltage and current of the coils of the magnets. The measurement was performed under different frequencies at room temperature.*
Data source location	*Wuhan University of Technology, Wuhan, China,*
Data accessibility	*Data is supplied with in this article.*

**Value of the data**

1. Electric polarization data was used to confirm the ferroelectricity of the La_0.2_Pb_0.7_Fe_12_O_19_ ceramics at room temperature.
2. Magnetic polarization data was used to demonstrate the ferromagnetism of the La_0.2_Pb_0.7_Fe_12_O_19_ at room temperature.
3. The variable dielectric constants upon magnetic field were used to display the giant magnetocapacitance effect of the La_0.2_Pb_0.7_Fe_12_O_19_ ceramics at room temperature.
4. The three datasets demonstrate not only the coexistence of large ferroelectricity and strong ferromagnetism in single La_0.2_Pb_0.7_Fe_12_O_19_ at room temperature, but also the realization of its strong magnetoelectric coupling effect at room temperature.

## 1 Data

The data given in this data article is in the form of three figures. It describes briefly the preparation process of the La_0.2_Pb_0.7_Fe_12_O_19_ ceramics, the electric and magnetic characterization of the La_0.2_Pb_0.7_Fe_12_O_19_ ceramics as well as its magnetoelectric coupling phenomenon. The data article includes ferroelectric and I–V datasets giving the information of the electric polarization and two current peaks upon applied field, ferromagnetic datasets and variable magnetic field dependent-capacitance datasets.

## 2 Experimental design, materials and methods

The single-phase La_0.2_Pb_0.7_Fe_12_O_19_ powders were prepared by a polymer precursor method, certain amount of power was pressed into a pellet which was sintered into ceramic. After sintering, the ceramic has subsequently experienced heat-treatment in O_2_ at 700 °C three times to remove the oxygen vacancies and transform Fe^2+^ into Fe^3+^ in the La_0.2_Pb_0.7_Fe_12_O_19_ ceramic. for P-E hysteresis loop measurement, both sides of the ceramic surfaces were coated with silver paste as electrode which was sintered at 820 °C for 15 min. Then the ferroelectric hysteresis loop was measured upon the polycrystalline La_0.2_Pb_0.7_Fe_12_O_19_ pellet with electrodes by using an instrument referred as ZT-IA ferroelectric measurement system. Magnetization measurement was carried out on a Quantum Design Physical Property Measurement System (PPMS). The magnetocapacitance parameters of the La_0.2_Pb_0.7_Fe_12_O_19_ ceramics were measured using a Wayne Kerr 6500B LCR station or an IM 3533-01 LCR meter by applying a variable magnetic field on the specimen.

## 3 Multiferroic characterization of La_0.2_Pb_0.7_Fe_12_O_19_ ceramics

### 3.1 Structure identification

**Fig. 1:**
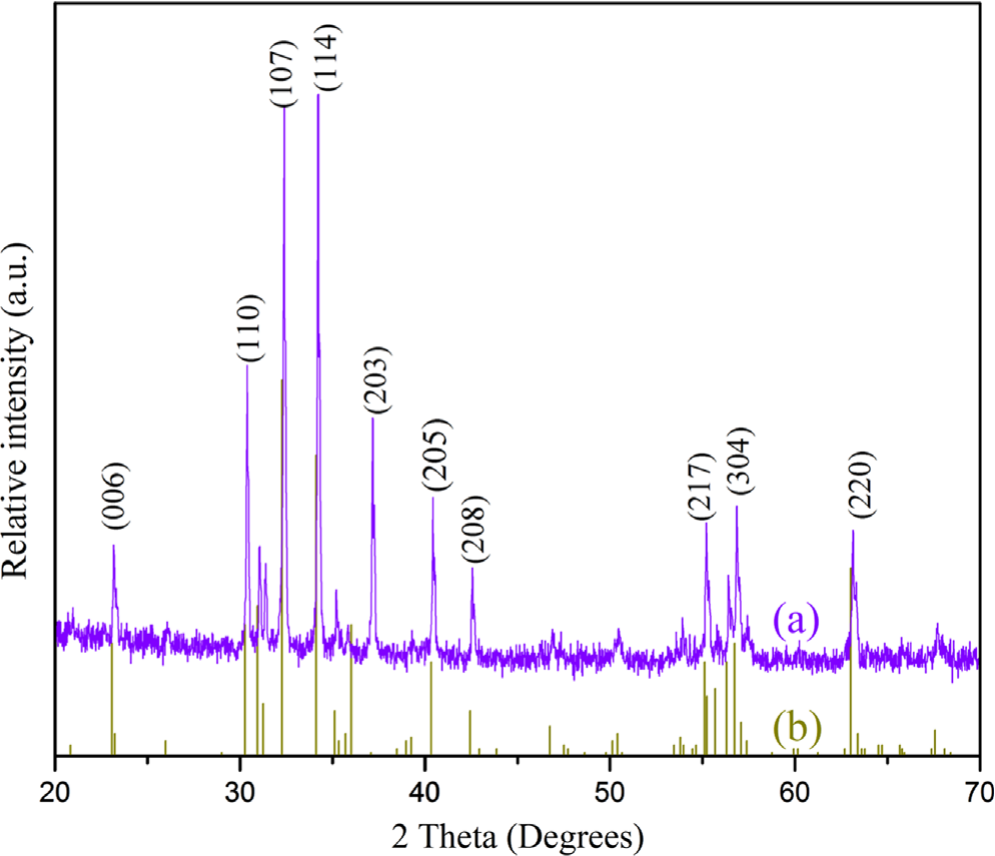
Structure identification of La_0.2_Pb_0.7_Fe_12_O_19_. (a) XRD pattern of the La_0.2_Pb_0.7_Fe_12_O_19_ with O_2_ annealing process. (b) The standard diffraction pattern of the PbFe_12_O_19_ compound (PDF#15–0623) being marked by red discrete lines. (For interpretation of the references to color in this figure legend, the reader is referred to the web version of this article).

Fig. 1a shows XRD pattern of O_2_ treated La_0.2_Pb_0.7_Fe_12_O_19_ ceramics; the underneath red diffraction lines are coming from the standard diffraction spectrum of PbFe_12_O_19_ (PDF#15–0623). The CIF file from Rietveld refinement has been included in this article. The La_0.2_Pb_0.7_Fe_12_O_19_ ceramics with oxygen treatment is identified to be magnetoplumbite-5H structure, which is the same structure of PbFe_12_O_19_. The Rietveld refinement results suggest that La atoms have been successfully replaced Pb atoms in PbFe_12_O_19_ and has entered into its crystal lattice. The lattice unit has been contracted about 0.44% after the replacement of Pb atoms with La atoms. The overlap of the two diffraction patterns reveals that La_0.2_Pb_0.7_Fe_12_O_19_ shares the same crystal structure with PbFe_12_O_19_.

### 3.2 Ferroelectricity and I–V features

**Fig. 2:**
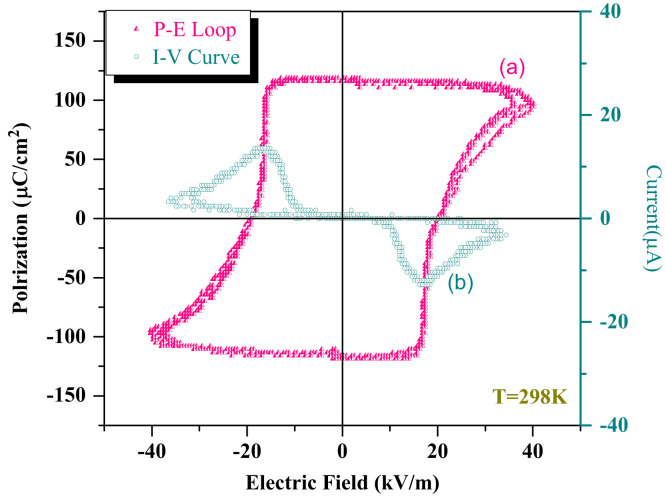
Ferroelectric characterization of La_0.2_Pb_0.7_Fe_12_O_19_: (a) The ferroelectric hysteresis loop of La_0.2_Pb_0.7_Fe_12_O_19_ ceramic being measured at a frequency of 33 Hz and 298 K; (b) the nonlinear I–V curve of La_0.2_Pb_0.7_Fe_12_O_19_ ceramic. The ceramic was sintered at 1000 °C for 1 h and subsequently heat-treated in an O_2_ atmosphere for 9 h. The measurement was performed at room temperature.

Fig. 2a displays a electronic polarization hysteresis loop of La_0.2_Pb_0.7_Fe_12_O_19_ ceramics with full saturation. The ferroelectric properties were measured upon a lab-constructed ZT-I system at a frequency of 33 Hz and 298 K. The polarization loop looks like that of most standard ferroelectric compounds. The remnant polarization of the hysteresis loop is about 119.4 μC/cm^2^. This value is a little higher than that of pure PbFe_12_O_19_ ceramic (104 μC/cm^2^) [1] obtained by the same process. The coercive field of the La_0.2_Pb_0.7_Fe_12_O_19_ ceramics is determined to be 20.6 kV/m.

Fig. 2b demonstrates two I–V peaks at the vicinity of positive and negative coercive field. The two particular peaks present a great nonlinear relationship between voltage (V) and current (I), which provides us with an additional evidence for the ferroelectricity of La_0.2_Pb_0.7_Fe_12_O_19_ ceramics and could exclude the possibility of current leakage. The classic ferroelectric hysteresis loop with full saturation and two nonlinear I–V peaks convinced us that La_0.2_Pb_0.7_Fe_12_O_19_ is indeed a ferroelectric compound. The origin of the ferroelectricity of M-type lead hexaferrite has been discussed in detail in the previous literatures [1], [2].

### 3.3 Ferromagnetism

**Fig. 3:**
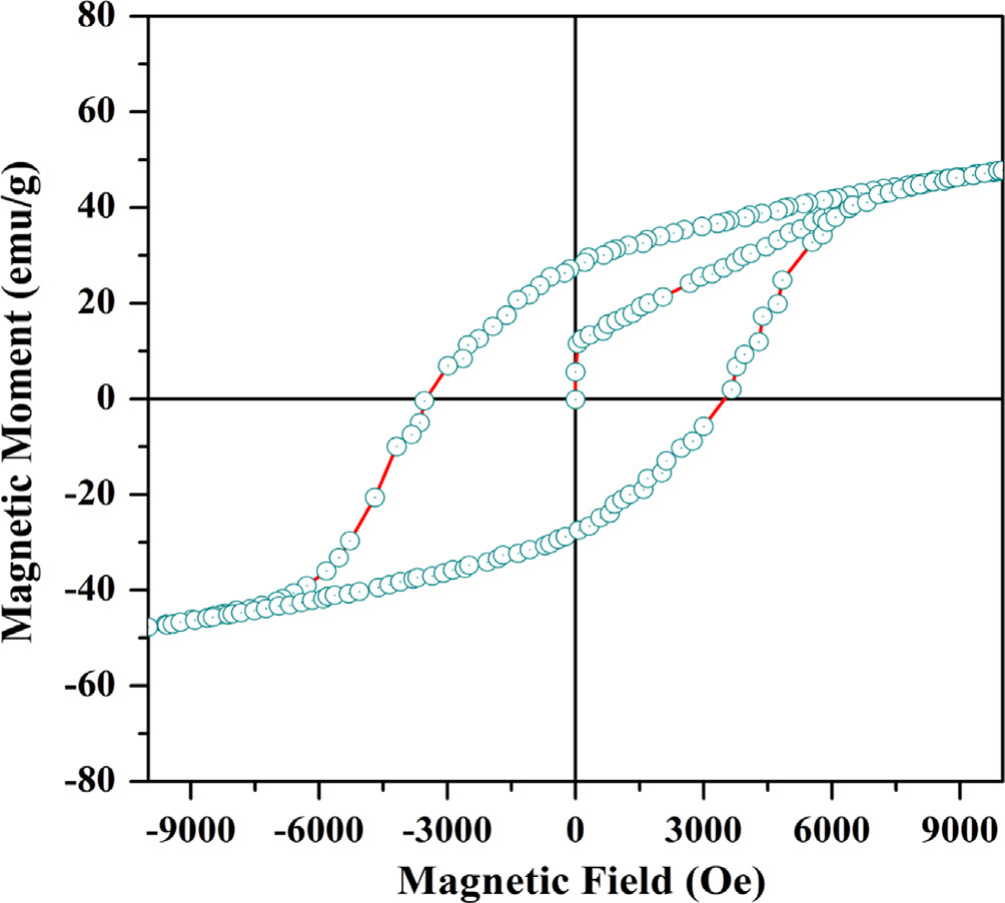
Magnetic hysteresis loop of polycrystalline La_0.2_Pb_0.7_Fe_12_O_19_ ceramic being sintered at 1000 °C for 1 h and subsequently annealed in O_2_ for 9 h. The measurement was performed at room temperature.

Fig. 3 demonstrates the magnetization hysteresis loop of La_0.2_Pb_0.7_Fe_12_O_19_. The remnant magnetic moment (M_r_) is about 28.7 emu/g and the coercive magnetic field (H_c_) is around 3439.2 Oe. The remnant moment of La_0.2_Pb_0.7_Fe_12_O_19_ has been reduced by a small amount while its coercive field has been enhanced by 1114 Oe in comparison with that of pure PbFe_12_O_19_ [1], since the substituted La provides additional unpaired f electrons, which create more magnetization moment. The large hysteresis loop reflects the strong magnetic feature of La_0.2_Pb_0.7_Fe_12_O_19_.

### 3.4 Giant magnetocapacitance effect

**Fig. 4:**
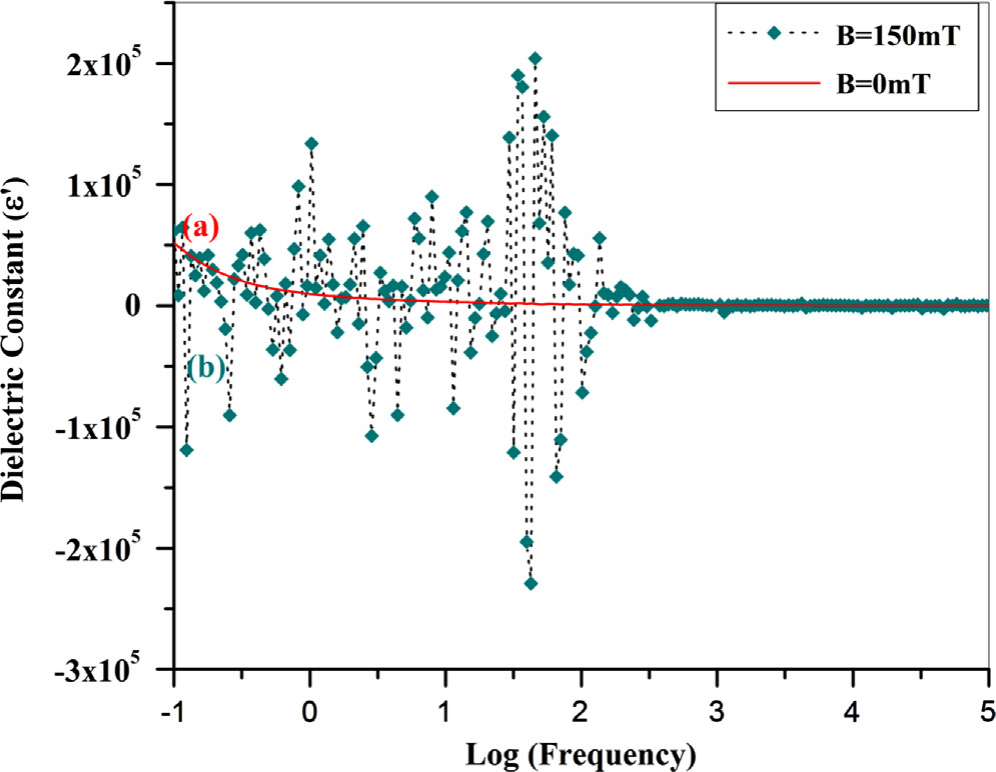
Frequency-dependent dielectric constant of La_0.2_Pb_0.7_Fe_12_O_19_ ceramics upon the magnetic field of (a) *B*=0 mT (red solid line) and (b) *B*=150 mT (dark cyan dotted line). The specimen was air-sintered at 1000 °C for 1 h and subsequently annealing in O_2_ for 9 h. The measurement was performed at room temperature. (For interpretation of the references to color in this figure legend, the reader is referred to the web version of this article).

In order to check out the magnetoelectric coupling effect of La_0.2_Pb_0.7_Fe_12_O_19_ ceramics, we then measured the capacitance of the specimens at different frequencies upon a applied magnetic field. The specimen was coated with silver electrodes on both surfaces and then put in a space between two magnets, the frequency-dependent capacity data was measured by a IM3533-1 LCR Meter (Hioki Company, Japan) without and with applying a magnetic field at *B*=150 mT. Fig. 4b shows the great response of capacity (or dielectric constant) to the magnetic field as a function of frequencies for La_0.2_Pb_0.7_Fe_12_O_19_ ceramics. We found that there was a giant response of the capacitance (dielectric constant) to the magnetic field at low frequency region. There is big difference between the dielectric constants without (Fig. 4a) and with (Fig. 4b) external magnetic field. Upon the applied magnetic field (*B*=150 mT), the dielectric constant (ε(*B*)) could oscillate along with ε(0) in a great amplitude, varying from 204065.6 to −229257.9. The dielectric constant (ε(*B*=150 mT)) was determined to be 68184 at the frequency of 50 Hz and 42951 at 100 Hz, respectively (Fig. 4b); while the corresponding dielectric constants at *B*=0 (ε(0)) equal to 921.4 at 50 Hz and 731.6 at 100 Hz, respectively. The maximum value of the magnetocapacitance ratio exceeds 2.18×10^4^% (Δε(B)=(ε(B)−ε(0))/ε(0), *B*=150 mT and *f*=50 Hz). These values match well with that being measured by a Wayne Kerr 6500B LCR station [3]. The agreement between two sets of capacitance data (*B*=150 mT) being measured from two different LCR meters indicates the reliability and repetitiveness of the B-field dependent dielectric constants.
