# Supplementary material for: Multiferroic La0.2Pb0.7Fe12O19 ceramics: Ferroelectricity, ferromagnetism and colossal magneto-capacitance effect
Source: Data Brief. 2016 Nov 24;10:69–74. doi: 10.1016/j.dib.2016.11.067 (PMC5137171; doi:10.1016/j.dib.2016.11.067)
Supplement: Supplementary file 1 — Supplementary material [file mmc1.docx]

Conflict of interest form

There is no conflict of interest.
