# Supplementary figures and images for: Multiferroic La0.2Pb0.7Fe12O19 ceramics: Ferroelectricity, ferromagnetism and colossal magneto-capacitance effect
Source: Data Brief. 2016 Nov 24;10:69–74. doi: 10.1016/j.dib.2016.11.067 (PMC5137171; doi:10.1016/j.dib.2016.11.067)

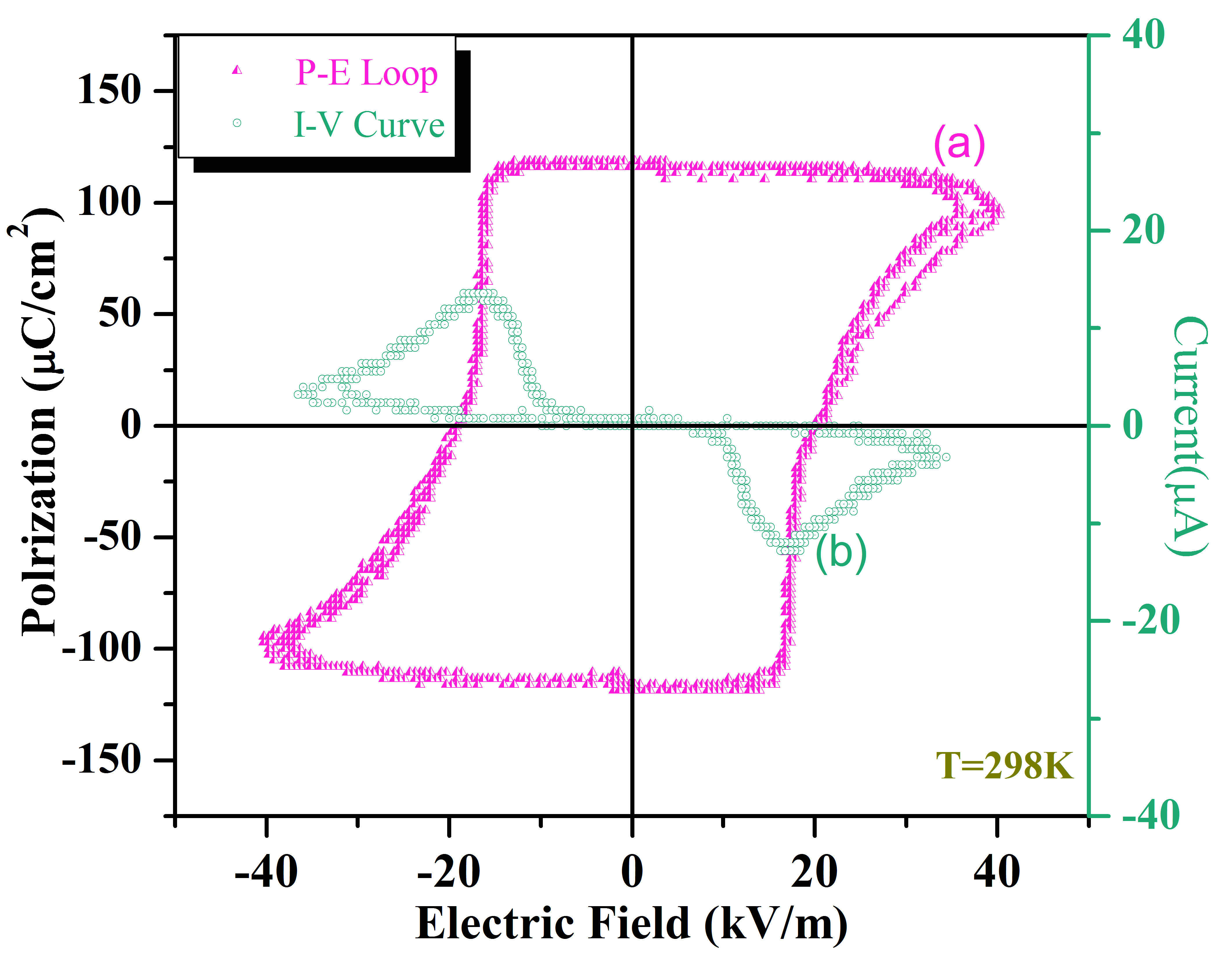

Supplement: Supplementary file 2 — Supplementary material [file mmc2.zip › LPFO PE+IV DIB.tif]

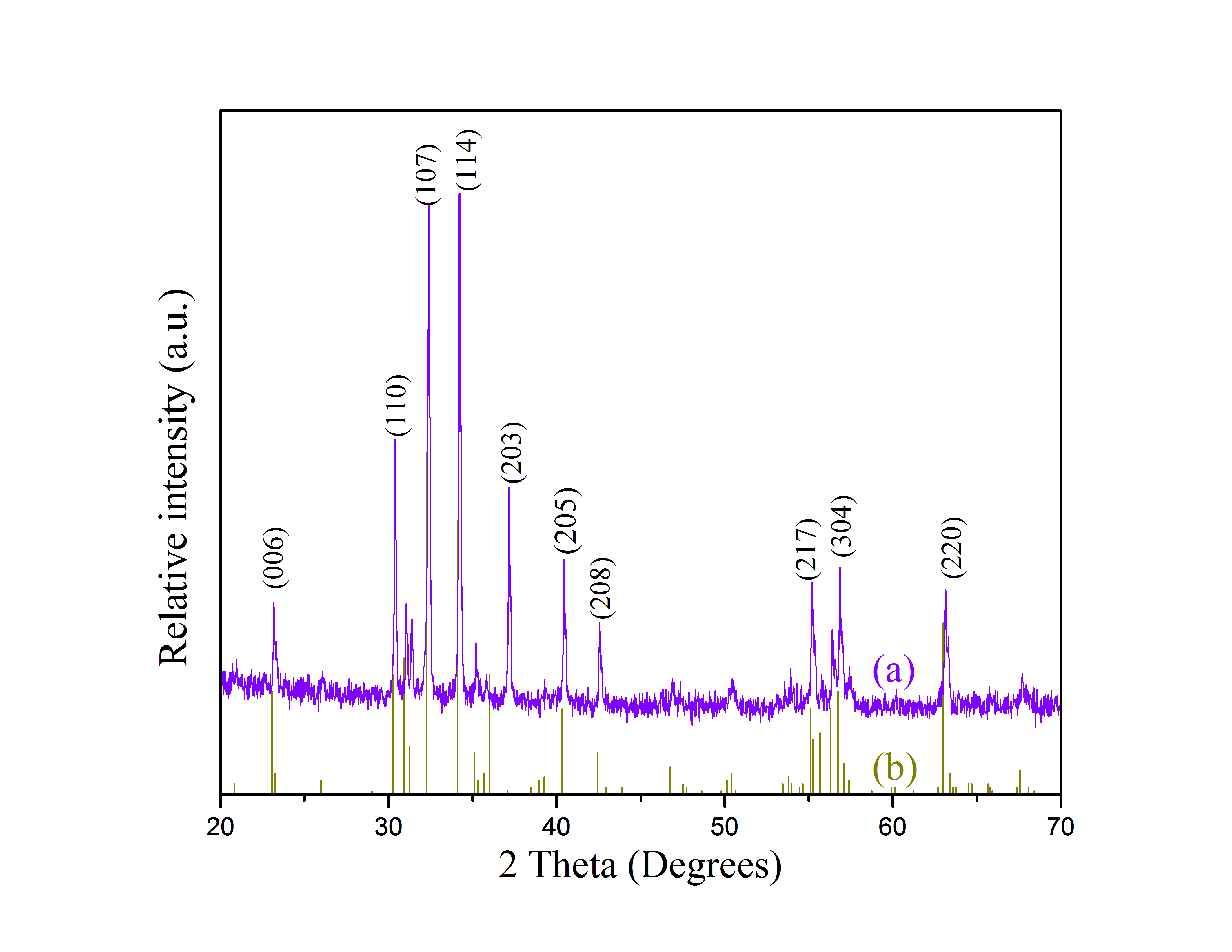

Supplement: Supplementary file 2 — Supplementary material [file mmc2.zip › LPFO XRD.tif]

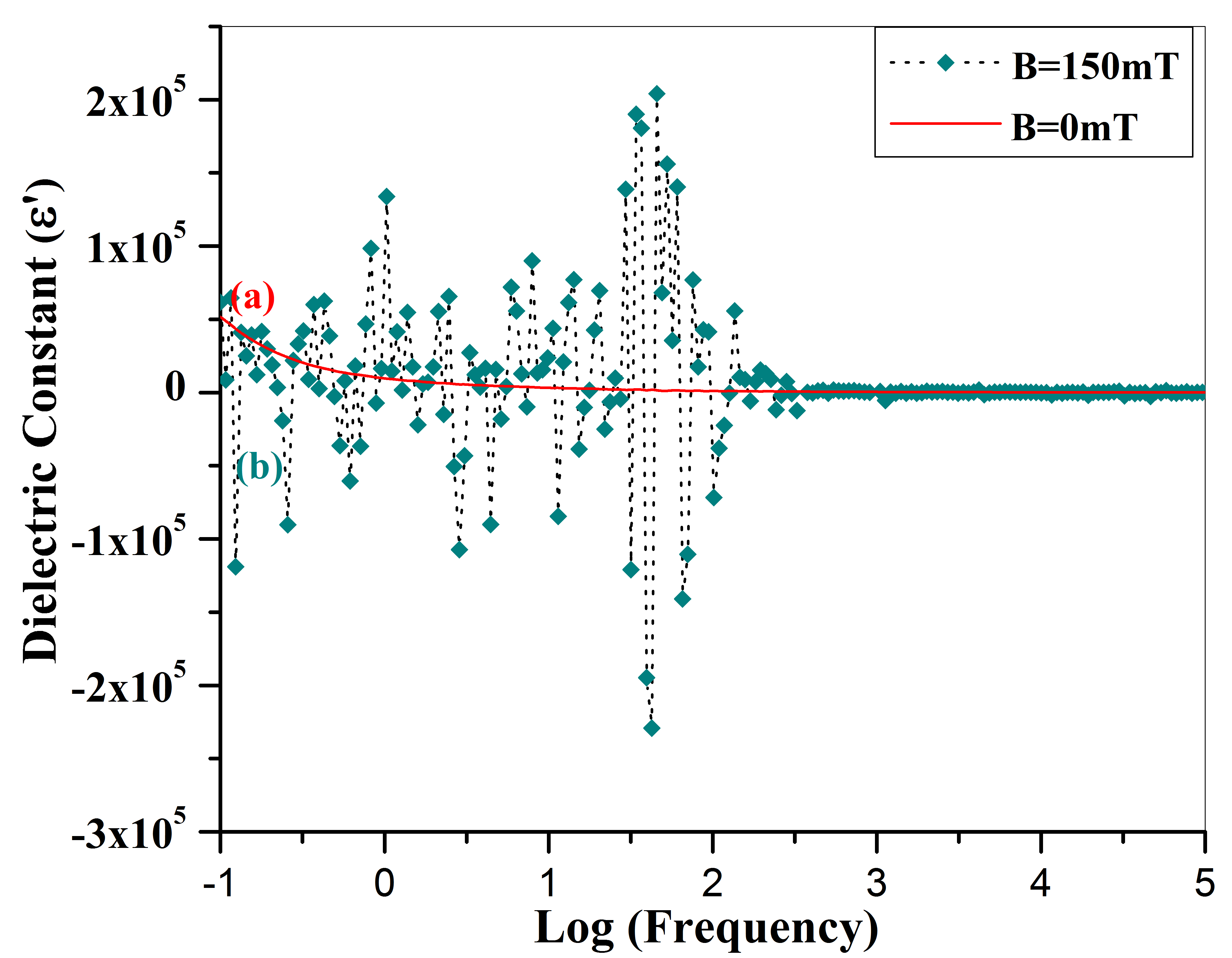

Supplement: Supplementary file 2 — Supplementary material [file mmc2.zip › LPFO ef+B.tif]

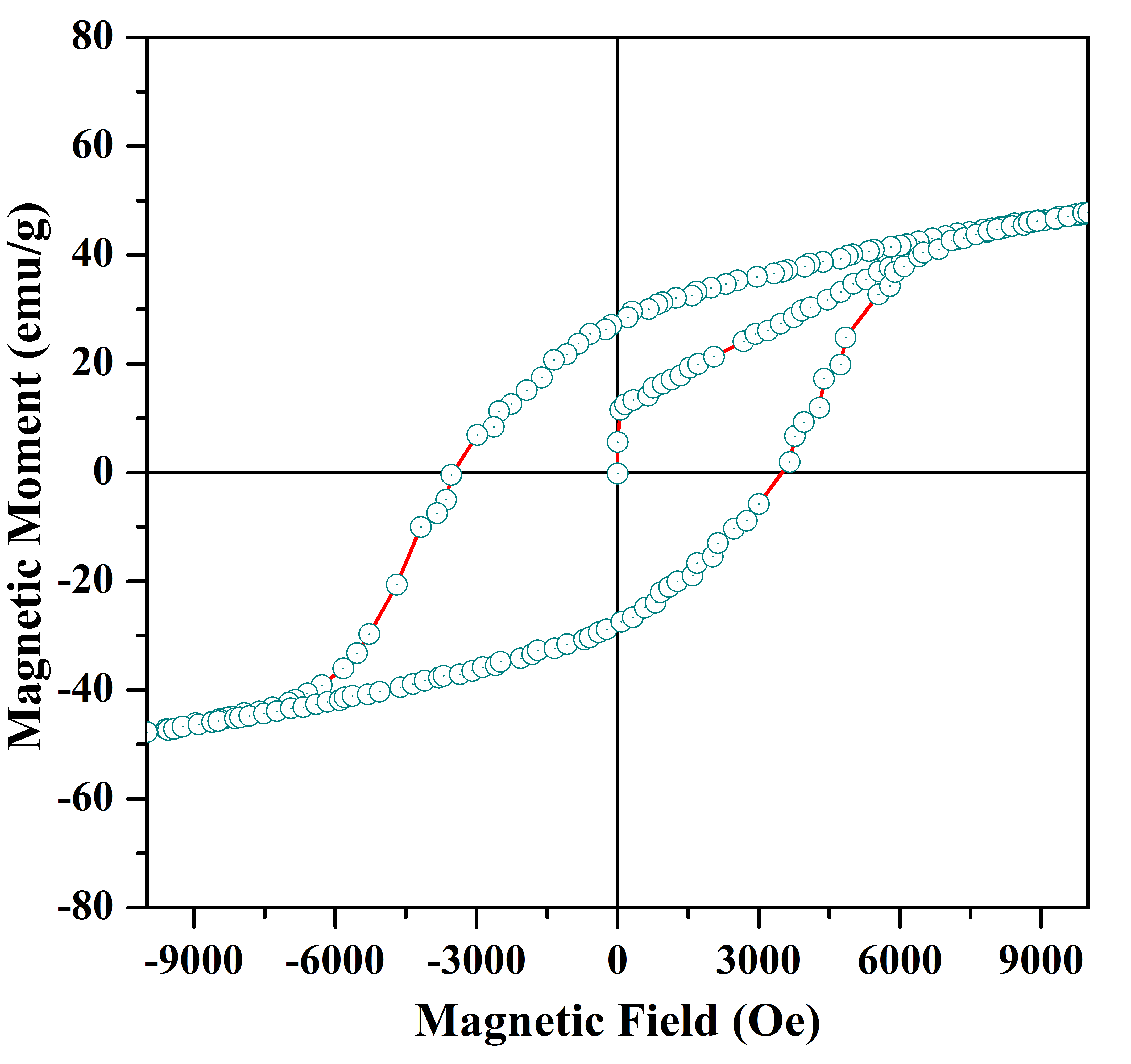

Supplement: Supplementary file 2 — Supplementary material [file mmc2.zip › LPFO MH Loop.tif]
